# Co-Design of an eHealth Intervention to Reduce Cardiovascular Disease Risk in Male Taxi Drivers: ManGuard

**DOI:** 10.3390/ijerph192215278

**Published:** 2022-11-18

**Authors:** James McMahon, David R. Thompson, Kevin Brazil, Chantal F. Ski

**Affiliations:** 1 School of Nursing and Midwifery, Queen’s University Belfast, Belfast BT9 7BL, UK; 2 Integrated Care Academy, University of Suffolk, Ipswich IP4 1QJ, UK

**Keywords:** eHealth, co-design, cardiovascular disease, men, taxi drivers

## Abstract

Taxi driving, a male-dominated occupation, is associated with an increased risk of cardiovascular disease (CVD). The increased risk is linked to a high prevalence of modifiable CVD risk factors including overweight/obesity, poor nutrition, smoking, excessive alcohol consumption and physical inactivity. Behaviour change interventions may prove advantageous, yet little research has been conducted to reduce CVD risk in this population. The purpose of this study was to co-design an eHealth intervention, ‘ManGuard’, to reduce CVD risk in male taxi drivers. The IDEAS framework was utilised to guide the development of the eHealth intervention, with the Behaviour Change Wheel (BCW) incorporated throughout to ensure the intervention was underpinned by behaviour change theory. Development and refinement of ManGuard was guided by current literature, input from a multidisciplinary team, an online survey, a systematic review and meta-analysis, and focus groups (*n* = 3) with male taxi drivers. Physical inactivity was identified as the prime behavior to change in order to reduce CVD risk in male taxi drivers. Male taxi drivers indicated a preference for an eHealth intervention to be delivered using smartphone technology, with a simple design, providing concise, straightforward, and relatable content, and with the ability to track and monitor progress.

## 1. Introduction

Globally, cardiovascular disease (CVD) remains the leading cause of mortality and morbidity [1]. Men are at a higher risk of developing the disease compared to their female counterparts [2,3]. Taxi driving, a male-dominated occupation [4], is associated with an increased risk of CVD, with long working hours linked to sustained psychological stress [5]. Taxi drivers also present with greater levels of CVD-related risk factors and behaviours, including high blood pressure, overweight and obesity, cigarette smoking, overconsumption of alcohol, a lack of fruit and vegetables, and prolonged periods of sedentary behaviour [5,6,7,8,9,10]. 

Despite the well-reported risk of CVD associated with the occupation [5,7,9,11,12,13,14,15,16,17,18], little has been done to reduce their risk. To our knowledge, only two interventions aimed at bringing about behaviour change in this population have undergone testing [13,19]. Both interventions aimed to improve physical activity through assessing change in walking-related outcomes. Encouragingly, both interventions produced positive results, with one study reporting significant improvements for all walking-related measures [19]. However, the other study [13] reported a high attrition rate (61%).

One way to improve intervention uptake, adherence, and subsequent success is through electronic health (eHealth) [20]. eHealth involves the use of information and communication technologies to support health and healthcare delivery, allowing interventions to be tailored to meet the needs of the end-user. eHealth interventions utilise technology for “intervening in an existing context by changing behaviour and/or cognitions” [20]. Key benefits of eHealth include an ease of access and usability for the user [21]. This may prove advantageous for male taxi drivers who present as a hard-to-reach group due to the remote nature of the occupation. Recent systematic reviews and meta-analyses have reported the success of eHealth interventions for promoting behaviour change in men [22,23] and those living with non-communicable diseases [24]. However, eHealth is not without its weaknesses, including poor intervention adherence and high rates of attrition [25,26]. Such issues are commonly attributed to a lack of consideration towards the relationship between the technology and target user during the development process [20]. 

Co-design, which involves an ongoing collaboration between researchers, developers, and end-users [27], allows for the development of an intervention that will be better accepted by the intended user. Co-design has been recommended for the development of interventions targeting men [28], though this rarely occurs. For example, a recent systematic review of 36 interventions that assessed weight as an outcome, reported that only nine studies consulted with target users during the development process [29]. Utilising an iterative approach between developers and end-users throughout the development process is crucial for the success of an eHealth intervention [30]. Additional factors deemed necessary to ensure the success of an eHealth intervention include being grounded in behaviour change theory, developed with an in-depth understanding of the target population, evaluated rigorously, and disseminated widely [31]. There is currently a lack of frameworks available for guiding the development of effective eHealth interventions [30,32,33,34], none of which consider all the aforementioned factors deemed necessary for an effective intervention. Thus, the IDEAS (Ideate, DEsign, Assess, and Share) framework was developed [31], successfully utilised for the development of an eHealth intervention for improving vegetable consumption in overweight adults [35].

In line with co-design, IDEAS draws on a human-centred design approach, through developing an understanding of the intervention’s end-users to ensure their needs are met [20]. The framework is split into four overarching phases: Integrate, Design, Assess, and Share. Across these phases are ten individual phases: (1) empathize with target users; (2) specify target behaviours; (3) ground in behavioural theory; (4) ideate implementation strategies; (5) prototype potential products; (6) gather user feedback; (7) build minimum viable product; (8) pilot potential efficacy and usability; (9) Evaluate efficacy; and (10) share intervention and findings. The aim of this study was to co-design an eHealth intervention, ‘ManGuard’, to reduce CVD risk in male taxi drivers working in Northern Ireland.

## 2. Methods

Guided by IDEAS, ManGuard was developed through a robust co-design process, involving an iterative collaboration between interdisciplinary experts and end-users. This paper reports on the findings from phases 1–6 of the IDEAS framework, resulting in the development of a proof-of-concept (PoC) of the ManGuard intervention. A PoC is a theoretical demonstration of a product to ensure the idea is feasible and will function appropriately [36]. The PoC will be developed into a minimal viable product (MVP) (phase 7) to undergo pilot testing (phase 8), followed by a fully powered randomised controlled trial (RCT) (phase 9), if deemed feasible.

Reviews of eHealth interventions have reported an association between the inclusion of behaviour change theories and improved intervention effectiveness [37,38]. To provide a more comprehensive and systematic approach for the identification and inclusion of behaviour change theory, the Behaviour Change Wheel (BCW) [39] was incorporated throughout relevant phases of IDEAS. Utilising the BCW not only ensures the inclusion of behaviour change theory but helps intervention developers to identify the most effective intervention functions, policies, and behaviour change techniques (BCTs) for fostering successful behaviour change. The BCW is comprised of eight steps: (1) define the problem in behavioural terms; (2) select target behaviours; (3) specify the target behaviour; (4) identify what needs to change; (5) intervention functions; (6) policy categories; (7) behaviour change techniques; and (8) mode of delivery. Due to a lack of access to ‘policy levers’, e.g., government officials, step 6 was omitted [40]. In addition, as the aim of this study was to develop an eHealth intervention, identifying the mode of delivery (step 8) was not addressed. 

### 2.1. Phase 1: Empathize with Target Users

Aligning with step 1 of the BCW (define the problem in behavioural terms), phase 1 of IDEAS aims to develop a greater understanding of the target population and their behaviours, allowing for the development of a more creative and motivating intervention [31]. Phase 1 was achieved through two steps: (i) an online survey; and (ii) input from a project advisory group (PAG).

#### 2.1.1. Step One: Online Survey

Due to a lack of data available on taxi drivers working in Northern Ireland, an online survey was conducted to gain a greater understanding of the target population. Questions aimed to uncover their experiences of working as a taxi driver (work habits, lifestyle, and behaviours) and initial intervention preferences (format and content). The survey was conducted via two methods: (i) a Facebook group dedicated to male taxi drivers working in Northern Ireland (>1300 drivers); and (ii) through software used by those working for the country’s largest taxi company (>1200 drivers). The survey link was shared several times between September and November 2020 until responses ceased entirely. Survey responses were imported into Microsoft Excel to be assessed and reported descriptively.

#### 2.1.2. Step Two: Project Advisory Group

Step two involved the formation and first of four meetings of the PAG, an interdisciplinary team of eight members, comprised of multidisciplinary experts:

(i) A PhD student with expertise in clinical exercise physiology
(ii) A nurse with expertise in cardiovascular nursing and rehabilitation
(iii) A psychologist with expertise in cardiovascular care
(iv) A health services researcher with expertise in developing and evaluating health/social care interventions
(v) A computer scientist/software developer
(vi) A mental health researcher and member of the Men’s Health Forum in Ireland (MHFI)
(vii) Two male taxi drivers.

End-users involved in the PAG were sourced through a company memo, delivered directly to all drivers working for one of Northern Irelands largest taxi companies via software used by all employees. Those interested in taking part were instructed to contact JM using the email provided. Involving end-users from the outset has been reported to enhance the acceptability of the intervention, improving user engagement [41,42]. The aim of the first PAG meeting was to guide the initial development of solutions seen as relevant and important to the end-users in tackling CVD risk in taxi drivers and provide qualitative insights into life as a taxi driver in Northern Ireland. The meeting was conducted in-person, audio recorded, with field notes taken by a member of the research team. A summary of the key findings from the PAG meeting will be presented.

### 2.2. Phase 2: Specify Target Behaviours

Phase 2 of IDEAS encompassed step 2 (select the target behaviour) and 3 (specify the target behaviour) of the BCW. The aim of phase 2 was to identify the behaviour(s) that the intervention would aim to modify for the greatest reduction in CVD risk for male taxi drivers, and the context in which the behaviour(s) should occur. The BCW recommends taking a “less is more approach, focusing on changing one or two behaviours” [40]. Guided by the findings from phase 1 and a search of relevant literature, a ‘long list’ of behavioural risk factors for CVD in the target population was generated. The following criteria were used for assessing and selecting the most appropriate behaviour(s) [40]:

- **Impact of behaviour change:** The likely impact that changing the behaviour could have on the population;
- **Likelihood of changing behaviour:** The likelihood of the population being able to change the behaviour (capability, opportunity, and motivation);
- **Spill over score:** The possible spill over each behaviour change could have on another behaviour;
- **Measurement score:** How easy will it be to measure change in the behaviour.

Next, the target behaviour(s) were specified by ascertaining the context in which it should occur for successful behaviour change to be achieved. Consideration was given to (i) who needs to perform the behaviour; (ii) what does the person need to do differently to achieve the desired change; (iii) when will they do it; (iv) where will they do it; (v) how often will they do it; and (vi) with whom will they do it [40].

### 2.3. Phase 3: Ground in Behavioural Theory

Phase 3 of IDEAS ensured the development of the intervention was grounded in behavioural theory, encompassing steps 4, 5, and 7 of the BCW. First, a behavioural analysis was conducted to identify the individual and environmental factors in need of modification to improve the likelihood of successful behaviour change occurring (step 4 of the BCW; identify what needs to change). The behavioural analysis was conducted using the findings from phase 1 and 2 of IDEAS, as well as existing literature, to aid a greater understanding of the taxi drivers’ capabilities, opportunities, and motivations. The BCW suggests that an individual needs to possess the capability, opportunity, and motivation to successfully change a behaviour (COM-B model) [40].

**Figure 1:**
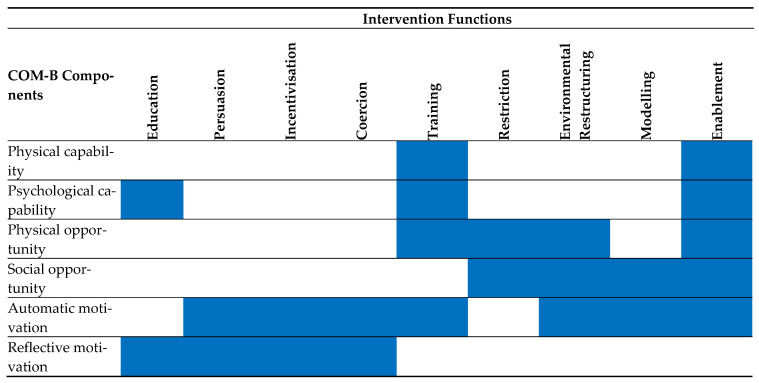
Matrix of links between COM-B and intervention functions, adapted from the. BCW handbook [39].

Next, intervention functions that could foster behaviour change were identified (step 5 of the BCW; identify intervention functions). Intervention functions are defined as “broad categories of means by which an intervention can change behaviour” [40]. The identified COM-B components were mapped to potential intervention functions using the ‘matrix of links’ (Figure 1) [40]. Utilising the Behaviour Change Technique Taxonomy (V1) [43], the final step of this phase (step 7 of the BCW; identify behaviour change techniques) was to identify and map appropriate BCTs to aid the implementation of the intervention functions. Guided by the BCW handbook [40], BCTs classified as “used most frequently” were initially considered before assessing those used less frequently. In addition, the systematic review and meta-analysis on the effectiveness of eHealth interventions for reducing CVD risk in men [22], was revisited to consider BCTs used in prior interventions targeting men.

### 2.4. Phase 4. Ideate Implementation Strategies

The remaining three phases of IDEAS addressed in this study (phases 4–6) are recommended to occur iteratively, involving input from an interdisciplinary team (PAG) and end-users for the development and refinement of the intervention [31]. To ensure readability, the processes involved in conducting these phases have been described as a linear approach. Three meetings were held online throughout the ideation phase. The first meeting aimed to gather feedback on the outcomes of phases 2 and 3. The second was to gather feedback on initial ideas for intervention content and design. Feedback was then used for modifications to be made to both content and design, displayed to the PAG for the third and final meeting. Email correspondences with relevant members of the PAG were used throughout the remainder of the development process when necessary.

### 2.5. Phase 5. Prototype of Potential Products

Prototypes provide an opportunity to test potential intervention content, design, and associated interactions [44], whilst gathering feedback from end-users to improve acceptability [41]. Low-fidelity prototypes were used for the development and refinement of ManGuard, offering a cost-effective method and greater ease of iteration [44]. Prototypes were shown to the PAG during the second and third meeting mentioned in the previous section (Section 2.4) to gather feedback and refine the intervention prior to progressing to phase 6.

### 2.6. Phase 6. Gather User Feedback

Three semi-structured online focus groups were conducted to gather end-user feedback for the refinement of intervention prototypes and content, with each focus group discussing different content. Questions focused on gaining feedback on language used, design, and anticipated navigation, steered by a topic guide developed by the research team. Recruitment was conducted online using the same methods used for conducting the online survey during phase 1. Focus group one aimed to gather feedback on minimal sketches of the anticipated layout and design of the intervention, as well as content for one module. Modules would provide educational materials in the form of ‘tips and tricks’, utilising motivational language to promote behaviour change. Focus group two aimed to display refinements made following the first session, in addition to content for a second module. The third and final session began with a display of refinements made following focus group two, and content for the remaining modules of the intervention. The same participants were involved during each focus group, with a £25 voucher provided for each session as a financial incentive.

**Figure 2:**
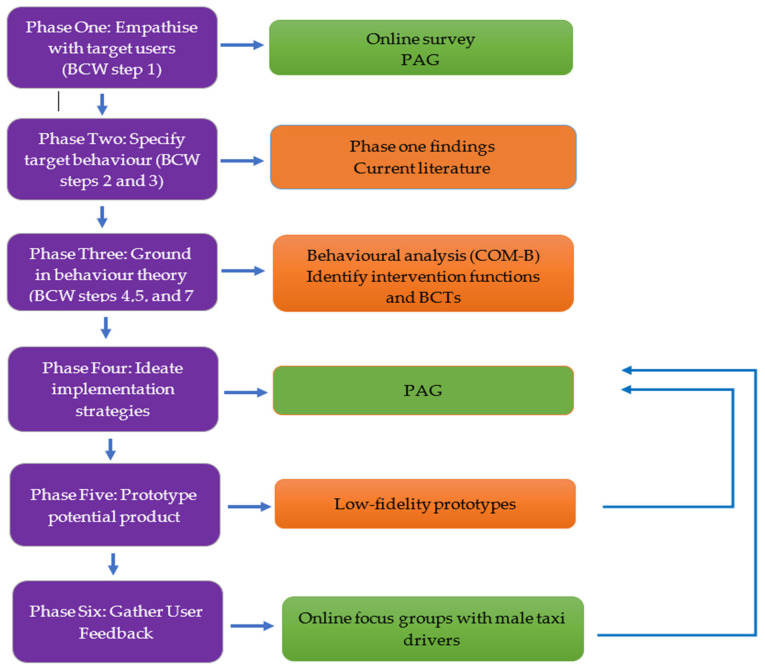
Illustrative overview of the study methods.

Focus groups were conducted online using the Microsoft Teams platform, with each session audio and video recorded, and then transcribed verbatim using Microsoft Word. Transcripts were analysed using an inductive approach through conventional content analysis [45]. As the focus of the analysis was on manifest data, category development was deemed appropriate as the highest level of abstraction [46,47,48]. Transcripts were analysed by a member of the research team (JM), with codes reviewed by a second member (KB) to ensure consensus and consistency. Figure 2 provides an illustrative overview of the study methods. 

## 3. Results

### 3.1. Phase 1: Empathize with Target Users

#### 3.1.1. Step One: Online Survey

A total of forty responses were retrieved from the online survey. Thirteen respondents were aged 30–40 years, ten between 40–50 years, and fifteen between 50–60 years. Two respondents were above 60 years of age. Respondents had a mean height and weight of 176.6 cm and 97.6 kg, respectively (average BMI = 31.2 kg/m^2^). On average respondents had been working for 10.8 years. Respondents were working an average of 5.7 days per week, for 9.6 h per shift, spending an average of 8.4 h seated during each shift. 

Two-thirds (67.5%) of respondents were not completing the recommended levels of physical activity, with most dietary habits rated between ‘average’ and ‘very poor’. One quarter of respondents were cigarette smokers. The overconsumption of alcohol did not appear to be of considerable concern, with one quarter presenting as current smokers, and an average alcohol consumption of 14.6 units/week. Knowledge of CVD was lacking, with over half (57.5%) of respondents knowing ‘a little’ about the condition. Physical inactivity and smoking were perceived by respondents as posing the greatest risk to their heart health, accounting for a quarter of responses each. 

Three-quarters (74%) of respondents were interested in engaging in an eHealth intervention to reduce their risk of CVD, with smartphones deemed the most appropriate mode of delivery (85%). Preferences for intervention length varied greatly between 4–10 weeks, with intervention interaction between 1–3 times per week. Almost half of respondents (47.5%) indicated a preference for each session to last ten minutes. Suggestions for improving participant motivation included progress tracking, reminders, discussing the intervention with others, and observing physical improvements.

#### 3.1.2. Step Two: Project Advisory Group

High stress levels were highlighted as an area of significant concern for taxi drivers working in Northern Ireland, caused by financial pressures, long working hours, and abusive customers. High levels of stress were stated to have a direct impact on the likelihood of engaging in risky behaviours such as overconsuming alcohol and gambling. Specific to Northern Ireland is the impact of ‘The Troubles’, a conflict that lasted more than three decades. Although the conflict ended more than 20 years ago, taxi drivers continue to feel unsafe transporting customers to certain areas, adding to their overall levels of stress. Loneliness and a lack of support were also mentioned as consequences of the job. Dietary habits were identified as a lifestyle habit in need of modification, with recommendations for healthy swaps whilst on the road being most appropriate. 

Module length was highlighted as being insignificant if the user had the ability to pause and re-access the content in their own time. However, session length should be no longer than 10–15 min for improved user engagement. It was recommended to keep concepts broad, and to utilise a straightforward and simplistic approach of delivery. The inclusion of gamification was of little interest unless a financial reward was involved due to a lack relationships between drivers. 

### 3.2. Phase 2: Specify Target Behaviours

Guided by a search of previously published literature, and the findings from phase 1, the common CVD-related risk behaviours associated with taxi driving were identified: physical inactivity, poor dietary choices, overconsumption of alcohol, and cigarette smoking. Physical activity was deemed the most appropriate behaviour to target to reduce CVD risk of male taxi drivers (Table 1).

Next, it was necessary to specify the target behaviour and the context in which it should occur for behaviour change to be achieved (Table 2). Recent guidelines from the World Health Organization [49] for physical activity and sedentary behaviour have signified a shift towards the completion of physical activity in bouts of any duration, with the aim of a promoting a more active way of living [50]. Therefore, no specific physical activity targets were set. It was determined that male taxi drivers should strive to complete more daily physical activity, whenever they can, wherever deemed suitable, and with whoever they choose.

### 3.3. Phase 3: Ground in Behavioural Theory

The behavioural analysis revealed that changes could be made to four of the six COM-B components to improve the target behaviour: psychological capability, social opportunity, and both reflective and automatic motivation. The identified components, alongside a description of what needs to occur for successful behaviour change, and why, can be found in Table 3.

Of nine intervention functions, seven were selected to facilitate change in the identified COM-B components: education, enablement, environmental restructuring, training, incentivisation, modelling, and persuasion. As discovered during the previous phases, knowledge of CVD, the positive impact of physical activity on CVD risk, and how to be active, are lacking. Thus, education was deemed appropriate for improving psychological capability and reflective motivation. Training and enablement were identified as functions to further improve psychological capabilities, imparting skills on how to incorporate physical activity into their daily routine, whilst helping to remove barriers to engaging in the behaviour.

Due to the lonely and isolated nature of the job, enablement, modelling, and environmental restructuring, could enhance their social opportunities. To enhance reflective motivation, education and persuasion were identified as having the potential to improve beliefs regarding the benefits of physical activity. Lastly, to enhance the automatic motivation of taxi drivers, persuasion, incentivisation, and training could stimulate a positive attitude towards being physically active.

Next, BCTs to successfully deliver the intervention functions were identified. As the Behaviour Change Technique Taxonomy (V1) is comprised of 93 individual BCTs, they are organised into 16 groupings. Ten groupings were identified as containing BCTs suitable for inclusion in ManGuard: (1) goals and planning; (2) feedback and monitoring; (3) social support; (4) shaping knowledge; (5) natural consequences; (6) comparison of behaviour; (7) repetition and substitution; (8) reward and threat; (9) comparison of outcomes; and (10) antecedents. Examples of the specific BCTs mapped to the intervention functions and COM-B components can be found in Table 4.

### 3.4. Phases 4 and 5: Ideate Implementation Strategies and Prototype of Potential Products

Due to the iterative process between ideation, prototype development, and refinement, the findings of phases 4 and 5 are presented together. During the first of three meetings of the PAG during these phases, a flowchart was displayed to present the core sections of ManGuard. Members of the PAG were satisfied with the core sections and the anticipated navigation. A social forum was proposed to allow taxi drivers to communicate with each other for improving social support. However, although agreed that it may prove beneficial, concerns of undesirable messages posted to the forum were raised. The provision of goal setting and progress tracking were confirmed as positive features. The taxi drivers recommended that users can assess and modify their goals over time to improve sustained engagement.

Guided by this feedback, content for one module, along with low-fidelity prototypes, were developed. During the second meeting, the taxi drivers emphasised the importance of all information being delivered in a simple, straightforward manner, and absent of technical jargon. The incorporation of positive and “punchy” wording, with images, was recommended to make the information more engaging. Although physical activity was identified as the most appropriate behaviour, taxi drivers may benefit from a holistic approach regarding the delivery of content due to the presence of several CVD-related risk factors. Thus, modules would be classified as either ‘core’ or ‘voluntary modules’. Core modules include: (1) heart disease, lifestyle, and goal setting; (2) physical activity; (3) nutrition; and (4) program re-cap. Voluntary modules include: (1) alcohol; (2) smoking; and (3) managing stress. It was recommended that users can access modules in an order they choose, except for the introduction and re-cap modules. Concerns were raised that a structured and linear approach may impact user satisfaction, engagement, and adherence.

Guided by this feedback, modifications were made to the low-fidelity prototypes and displayed during the third and final meeting of the PAG. A newly developed design for the homepage was well-received, perceived as straightforward and self-explanatory. The provision of positive welcome message at the start of each module was appreciated, recommended to be delivered using a pop-up feature to make it more attractive and engaging. Rather than a traditional graph for displaying user progress, it was recommended to use a “petrol gauge”, making the process more relatable to taxi drivers. It was agreed that physical activity would remain a primary option offered to the user for goals setting and progress tracking, with diet, smoking, and alcohol available as additional options. The inclusion of ‘awards’, collected by the user as they progress and achieve specific milestones, were perceived as a suitable method for the delivery of incentivisation and positive reinforcement.

### 3.5. Phase 6: Gather User Feedback

Six male taxi drivers, working in NI, were recruited to take part in three online focus groups. However, only four participants attended the first focus group. Following a lack of email responses between the first and second session, three participants were deemed to have withdrawn from the study. Therefore, three participants took part in the remaining two focus group sessions, lasting an average of 74 min. Four categories emerged from the analysis process: (1) program design; (2) program features; (3) program content; and (4) program engagement. A concise overview of key findings across the three focus groups are presented below under the associated categories.

#### 3.5.1. Program Design

Program design relates to comments made regarding aesthetic aspects of ManGuard, including the overall design and anticipated navigation. Participants highlighted the importance of ensuring a simple, straightforward, and self-explanatory design throughout.

*“Simpler the better.”*

[F1, P1]

Enhancing user experience is crucial for improving engagement. Participants perceived that having the ability to switch between a ‘light’ and ‘dark’ mode would improve readability and subsequent user experience.

*“You know what’s good as well, I like apps… during the night if I’m reading… the writing turns white and all the white that you have there is black which makes it easier to read… if it’s all lit up like, it’s more annoying.”*

[F1, P2]

To improve the process of goal setting, a ‘slider’ option was included in place of the user manually typing their goal. Although participants agreed that this would make the process more engaging and visually attractive, it was recommended to provide an option to type the goal as the slider may not be acceptable to all users.

*“So, would you also have an option for someone to type it in? ‘Cos some people aren’t very good with sliders? Sliders is definitely more visual, but if you have like, if you’re using your mobile phone for it and you’ve got arthritis or something it can be very hard using those slider things…”*

[F2, P1]

*“Yeah, I like it (slider), but as (P1) said, you can get some people who want to be really specific and you’re trying to get it right in the middle but struggling.”*

[F2, P2]

Between the first and second focus group, several logos for ManGuard were developed by a graphic designer. A design was chosen by the PAG, with all participants in agreement due to the plain, simple, and relatable design chosen, with “flashy” designs deemed less desirable.

*“I think it looks perfect. I think it looks clean, plain, it’s simple, you know, ManGuard, driving men’s wellbeing… like what else could you want? And then you’ve got a taxi logo there in the middle to act as an A as such, so no I think that’s perfect.”*

[F2, P2]

*“All black the way it is, white writing, suits me down to the ground, that’s simple and clean.”*

[F2, P3]

*“Yeah, I like that. I don’t like those flashy ones with loads of colour. It stands out well.*”

[F2, P1]

#### 3.5.2. Program Features

Program features relate to comments made on the characteristics of ManGuard, such as performance and functional capabilities. The utilisation of pop-ups with positive welcome messages at the start of each module was well received. Such a feature was highlighted as acting as an incentive to continue engaging with the module.

*“It makes it a bit more interesting I think than going onto a page that’s just full of static text… The pop-up thing is an added bonus to stay on the page, you know?”*

[F1, P2]

Concerns raised by the PAG members regarding the social forum, and it’s use for posting potentially offensive content, was further supported by the focus group participants.

*“Who’s going to monitor that messaging system?… All of us will know about taxi forums… there’s no concern to anybody… So, somebody needs to, you know, stop that.”*

[F1, P2]

In response to feedback regarding the social forum, a question and answer (Q&A) page was developed. During the testing of the MVP, this feature would allow users to message the research team, with the common queries and answers used to form a frequently asked question (FAQ) if ManGuard progressed beyond an MVP. Participants were in favour of this addition, perceiving this as a more acceptable option than the social forum.

*“What you’re saying is, if we have a problem that you would normally put on a forum for people, we would just send the question to you and… Yeah, probably better [than a social forum].”*

[F2, P1]

Awards, as a method of stimulating motivation and improved engagement, was not seen as a feature of great significance. It was highlighted that observing progress towards their goal would be sufficient motivation.

*“You know you’ve got one award, you might be a wee bit motivated to get two, but personally, I’d just be happy enough… if my target was 400 steps a day, if I hit that target that’s fine, you know what I mean?”*

[F1, P2]

Nonetheless, participants stated that it may prove motivational for others and recommended to retain awards in the final version.

*“I would keep them in because I know that there is people who like to see that stuff.”*

[F1, P1]

#### 3.5.3. Program Content

Program content relates to comments made on any information provided to the user, including text and imagery. Content across each page was recommended to remain concise to ensure users remained interested in the information. In addition, concise sections were deemed necessary due to the unpredictable nature of the job, allowing the user to better locate their place if they must leave the module due to work demands.

*“We have to remember its aimed at taxi drivers, so a lot of taxi drivers are going to be using this app in between jobs. So, you start reading something, then a job pops up. Say you have to go for straight away, so you don’t wanna be halfway down a three-page item that you lose your place when you come back to it… So nice short, concise paragraphs.”*

[F1, P1]

Following feedback from the PAG, imagery was incorporated throughout the content to accompany related information, highlighted as being appreciated by taxi drivers.

*“Or just put pictures in, taxi drivers love pictures.”*

[F1, P1]

Importantly, images should remain static, absent of links to external sites to prevent the user from becoming overwhelmed with information.

*“…if there’s too much to click, too much information… you can get lost… Keep the two things just as static pictures.”*

[F1, P1]

Feedback regarding clarity of information highlighted the need for minor modifications to be made. In particular, the phrase “we’re going to be with you all the way…” was deemed misleading and indicated that someone would be monitoring and providing direct advice throughout.

*“If you’re going to say we are going to be with you all the time… I mean, is somebody monitoring this in the background? Because if you’re not, you’re not with us all the way…”*

[F1, P2]

*“… that implies that you or somebody associated with you is going to be more or less monitoring what we’re putting in.”*

[F1, P1]

Incorporating content throughout that was relatable to taxi drivers was perceived to be a method of improving user satisfactions and engagement. Focus group participants agreed, specifically highlighting the relatability of the diet and physical activity content, respectively.

*“I think that’s extremely realistic… I think that’s bang on.”*

[F2, P3]

*“Have you been following me around or something? I eat and drink all of them.”*

[F3, P1]

Of significant importance was the feedback gathered on the module for coping with stress. It was indicated that due to the impact of the COVID-19 pandemic on the taxi industry, this module was more important now than ever.

*“Really, I would say that’s the most important one to be honest here.”*

[F3, P1]

*“Particularly over COVID, that one’s really brilliant… they were a forgotten industry and support… really were terribly supported.”*

[F3, P3]

#### 3.5.4. Program Engagement

The final category, program engagement, regards comments made on aspects of ManGuard that could both positively and negatively impact accessibility and usability of ManGuard. Financial incentives were stated as a tool that would stimulate motivation and sustained engagement.

*“…maybe, it’ll motivate us more if it was like when you get to level one you receive a £10 voucher, level 2 it’s £20…”*

[F1, P2]

Whilst engaging with the content, it was recommended to minimise the amount of ‘scrolling’ and ‘clicking’ throughout to prevent the user from losing interest and enhancing satisfaction.

*“… keep it as little as possible on each page and you have to click to go to the next one. If you’re scrolling, again me personally, I get bored and start flicking through it and miss sections.”*

[F1, P1]

The provision of informational pop-ups, discussed previously, were deemed as being a more interesting method of delivering content, providing an incentive to remain on the page and engage with the content.

*“It makes it a bit more interesting I think than going onto a page that just full of static text with maybe the odd video… The pop-up thing is an added bonus to stay on the page you know?”*

[F1, P2]

## 4. Discussion

This paper describes the robust step-by-step development, guided by the IDEAS framework, of an eHealth intervention (ManGuard) to reduce CVD risk in male taxi drivers. Additionally, to ensure the development of an intervention underpinned by behaviour change theory, the BCW was incorporated throughout the relevant phases of IDEAS. Intervention development involved an iterative process, guided by current literature, input from a multidisciplinary team (PAG), an online survey, a systematic review and meta-analysis, and focus groups with end-users. 

Survey responses support reports of male tax drivers engaging in higher levels of CVD-related risk behaviours. Such behaviours include physical inactivity [5,7,11,15,51] and poor dietary habits [51,52], and a high prevalence of cigarette smoking compared to the general population [53,54]. Interestingly, although the overconsumption of alcohol by taxi drivers has been regularly reported [9,16,52,54], survey responses indicate this as an area of little concern, with the average alcohol consumption only slightly higher than the recommended 14 units/week. However, it was stated by members of the PAG that many drivers would not admit to their regular alcohol intake. Therefore, these findings may be inaccurate, highlighting the potential issue of understanding the true alcohol habits of this population. 

It appears that although NI taxi drivers engage in greater levels of CVD-related risk behaviours, they are aware of the negative impact this has on their CVD risk. Interviews with taxi drivers report that the long shifts cause fatigue and a subsequent lack of energy for engaging in physical activity [52]. A lack of energy has been reported as a significant barrier for men engaging in physical activity [55]. Therefore, it is possible that male taxi drivers in Northern Ireland are physically inactive due to job-related fatigue. In addition, there appears to be a lack of health-related knowledge among this group. This is similar to findings from qualitative studies with taxi drivers, indicating a desire to be educated on healthy eating and physical activity to remove barriers towards successful behaviour change [15,52].

The online survey and input from the PAG further support previously reported associations between taxi driving and higher perceived levels of psychological stress [4,6,9,51,52,56,57,58]. Increased stress is caused by financial pressures, long working hours, and abusive customers. Interestingly, there appears to be a chain-reaction between several job-related factors. It was highlighted during the PAG meetings that financial pressures influence a belief of having to work longer hours, subsequently causing further stress. The increased level of stress then leads to further concern regarding finances, causing the cycle to continue infinitely. This chain-reaction has been referenced previously in a qualitative study of Hispanic taxi drivers in New York [52]. However, an additional factor specific to taxi drivers in Northern Ireland is the legacy of ‘The Troubles’. Taxi drivers continue to feel unsafe entering certain areas of the country, yet are pressured by their employers, only further adding to their levels of stress.

Although associations between stress and greater levels of engagement in CVD-related risk behaviours such as physical inactivity, poor diet, alcohol consumption, and smoking is well reported in the general population [59,60,61], associations are not as clear for taxi drivers. Of the limited studies that have explored the association between stress and CVD-related risk behaviours, two reported positive associations [6,53], one reported associations for smoking only [51], and another reported no association for any behaviours [58]. Nonetheless, the input of the taxi drivers in the PAG support those that reported a positive association, particularly for alcohol consumption. It was stated that many taxi drivers consume greater levels of alcohol as a means of dealing with stress. Future research aimed at improving levels of stress in male taxi drivers would prove beneficial. 

The online survey and conversations held with taxi drivers during PAG meetings helped uncover knowledge regarding the working lives of taxi drivers in Northern Ireland, data previously unexplored. Similar to previous research conducted with taxi drivers in other countries [5,9,13,14,56,57], those working in Northern Ireland had been in the job for an average of 11 years. Of concern is the significant association reported between working as a taxi driver for more than ten years and an increased risk of both CVD [9] and myocardial infarction [12]. This finding further supports the need, and potential benefit, of a behaviour change intervention to reduce the risk of CVD for male taxi drivers. The long working hours reported in the online survey (13 h shifts) are worrying due to prior research uncovering an association with this and greater levels of stress [58], consumption of psychoactive substances [54], and white blood cell counts [5] in this population, the latter being a marker for increased CVD risk. 

Findings from this study revealed the preferences of male taxi drivers for a novel eHealth intervention. Identified preferences of male taxi drivers in Northern Ireland are similar to those expressed previously by male taxi drivers in New York City, such as the intervention being delivered using smartphones (mHealth) [52], with a simple design, providing concise, straightforward, and relatable content [62]. Time constraints have been reported as having a major impact on the potential of male taxi drivers making positive behaviour change [62], further supported by the PAG. Therefore, to minimise potential impact on time, and in line with calls for concise content, information will be delivered in the form of ‘tips and tricks’, with imagery incorporated throughout. The provision of goal setting and tracking was well received by the male taxi drivers. There are no prior reports on male taxi driver preferences for goal setting. However, process evaluations of eHealth interventions targeted at male only samples have reported goal setting and progress tracking to be key intervention aspects [63], crucial for their success [64,65]. Additional program features well received by the participants of the focus group were ‘pop-ups’ to provide positive and motivational welcome messages, and awards for progressing through the program, acting as a tool for positive reinforcement.

### Limitations

There are some limitations to consider. First, and most important, was the participant attrition during the collection of qualitative data through online focus groups. Although risk management strategies were in place to reduce attrition, these were unsuccessful. As the study suffered from time-constraints due to the COVID-19 pandemic, it was not possible to delay data collection to allow for further recruitment to replace those that had withdrawn. Additional time may also have strengthened the study further, allowing for the conduction of qualitative interviewing during phase 1 to gain a further understanding of the target population. Second, although many taxi drivers expressed an interest in taking part in the study, recruitment to the online focus groups was poor. It is possible that taxi drivers dislike focus groups, or the online approach that was utilised due to COVID-19 restrictions. Future research should consider uncovering taxi driver preferences for engaging in research, including recruitment methods and data collection techniques.

## 5. Conclusions

This paper has reported on the utilisation of the IDEAS framework, incorporating the BCW throughout, for the co-design of an eHealth intervention (ManGuard) to reduce CVD risk in male taxi drivers working in Northern Ireland. Taxi driving as an occupation is associated with an increased risk of developing CVD. Yet, there are few interventions developed to reduce this risk, with poor participant attrition and intervention engagement reported. Co-design is one such way to develop an intervention that will be better accepted by the end-user. Male taxi drivers indicated a preference for an eHealth intervention delivered using smartphone technology, with a simple design, providing concise, straightforward, and relatable content, and with the ability to track and monitor progress. Next, in line with IDEAS, the PoC of ManGuard will be developed into an MVP to undergo feasibility testing (Phase 8). A protocol for the proposed feasibility trial has been published online [66].

## Figures and Tables

**Table 1:** Prioritising behaviours to reduce CVD risk in male taxi drivers.

Potential Target Behaviours Relevant to Reducing CVD Risk in Taxi Drivers	Impact of Behaviour Change ^a^	Likelihood of Changing Behaviour ^a^	Spill Over Score ^a^	Measurement Score ^a^
**Physical activity**	Very promising	Promising	Very promising	Very promising
**Dietary choices**	Very promising	Promising	Unpromising but worth considering	Promising
**Alcohol**	Promising	Unpromising but worth considering	Promising	Promising
**Smoking**	Very promising	Unpromising but worth considering	Promising	Promising

^a^ Rate as: unacceptable, unpromising but worth considering, promising, very promising.

**Table 2:** Specifying the target behaviour.

**Target Behaviour**	Male taxi drivers need to move more as a means of countering the sedentary nature of the job
***Who* needs to perform the behaviour?**	Male taxi drivers
***What* do they need to do differently to achieve the desired change?**	Move more every day (steps and/or moderate-vigorous intensity physical activity)
***When* do they need to do it?**	Everyday
***Where* do they need to do it?**	Home Work Community
***How often* do they need to do it?**	Everyday
***With whom* do they need to do it?**	Alone Friends Peers Family

**Table 3:** Behavioural analysis using the COM-B model.

COM-B Components	What Needs to Happen for Target Behaviour to Occur?	Is There a Need for Change?
**Psychological capability**	Know the risks accompanied with a lack of physical activity on CVD	Change needed due to findings from the online survey indicating a lack of knowledge, with most taxi drivers believing that other factors present the greatest risk towards their likelihood of developing CVD rather than physical inactivity
	Know what constitutes physical activity and the recommendations for how much they should aim to complete	Change needed due to survey responses demonstrating the belief that other behaviours are more pertinent to their risk of developing CVD, and therefore it is plausible that knowledge on the type/amount of physical activity needed to improve their risk is lacking
**Social opportunity**	Seeing others in close social networks being physically active	Change needed due to taxi drivers rarely seeing each other/spending time together as they work individually, as well as spending long hours at work away from family and friends, indicating a potential lack of social support
**Reflective motivation**	Hold beliefs that being physically active will help reduce their risk of developing CVD	Change needed due to many taxi drivers indicating a belief that behaviours other than physical activity are putting them at higher risk of CVD development
**Automatic motivation**	Create established routines and habits to be more physically active	Change is needed to be more physically active due to unpredictable nature of the job
**Behavioural analysis of the relevant COM-B components:**		Psychological capability, social opportunity, reflective motivation, and automatic motivation need to change for target behaviour to improve.

**Table 4:** Mapping the COM-B components and intervention functions to the corresponding BCTs.

Results of COM-B Analysis	Appropriate Intervention Functions	BCTs Utilised to Bring about Change (BCTv1) *	Example of How the BCT Could Be Applied to the Intervention
**Psychological Capabilities**	Education, Enablement, Training	2.2. Feedback on behaviour 3.1. Social support (unspecified) 4.1. Instruction on how to perform a behaviour 5.1. Information about health consequences	2.2. Inform the user on how many steps they have walked on a given day/week 3.1. Advise the user on seeking social support to make behaviour change and stick to the improved levels of physical activity 4.1. Provide the user with pictures and videos to explain how to complete the behaviour of being physical active 5.1. Advise the user on the benefits of the behaviour for CVD risk reduction
**Social opportunity**	Enablement, Modelling, Environmental restructuring	3.1. Social support (unspecified) 6.1. Demonstration of the behaviour 12.2. Restructuring the social environment	3.1. Provide the user with the ability to seek social support from peers through the program 6.1. Provide the user with information, pictures, and videos to demonstrate the completion of physical activity 12.2. Advise the user to change their social environment towards increasing the time spent with others who will also be physically active
**Reflective motivation**	Education, Persuasion, Enablement	1.1. Goal setting (behaviour) 1.2. Problem solving 1.3. Goal setting (outcome) 1.4. Action planning 1.6. Discrepancy between current behaviour and goal 2.2. Feedback on behaviour 2.3. Self-monitoring of behaviour 8.2. Behaviour substitution	1.1. Provide the user with the opportunity to set goals to achieve each week, e.g., how many steps to complete per day/week 1.2. Advise the user to analyse factors that influence their ability to complete physical activity and generate strategies to help them overcome perceived barriers 1.3. Provide the user the opportunity to set a goal to be achieved as a result of increasing their physical activity levels, e.g., setting a weight loss goal to assess change as a result of being more active 1.4. Advise the user to set a plan on when they will choose to be physically active including the frequency, duration, and intensity 1.6. Provide the user with feedback to make them aware that they are struggling to achieve the goal they had set for themself 2.2 Provide the user with automated feedback on their progress, e.g., how many steps they have completed that day 2.3. Provide the opportunity for the user to record and monitor their progress over time 8.2. Advise the user to go for a walk rather than sitting down on the sofa to watch tv or sitting in their car while waiting for a fare
**Automatic motivation**	Persuasion, Incentivisation, Training	8.3. Habit formation 9.1. Credible source 10.4. Social reward 10.6. Non-specific incentive	8.3. Advise the user to incorporate physical activity into their routine at the same time each day, e.g., plan to have a break at the same time on each working day and go for a walk during this time 9.1. Provide the user with credible/well known sources in favour of completing physical activity for reducing CVD risk 10.4. Provide the user with positive reinforcement messages when progress is achieved 10.6. Provide the user with positive reinforcement messages when progress is achieved and awards within the program, e.g., badges they can collect as the reach particular milestones

* The Behaviour Change Technique Taxonomy v1 [43].

## Data Availability

Data presented in this study are available on request from the corresponding author.
